# High-throughput single-cell sequencing of activated sludge microbiome

**DOI:** 10.1016/j.ese.2024.100493

**Published:** 2024-09-12

**Authors:** Yulin Zhang, Bingjie Xue, Yanping Mao, Xi Chen, Weifu Yan, Yanren Wang, Yulin Wang, Lei Liu, Jiale Yu, Xiaojin Zhang, Shan Chao, Edward Topp, Wenshan Zheng, Tong Zhang

**Affiliations:** a Environmental Microbiome Engineering and Biotechnology Lab, Department of Civil Engineering, The University of Hong Kong, Pokfulam Road, Hong Kong, 999077, China; b School of Public Health, The University of Hong Kong, Pokfulam Road, Hong Kong, 999077, China; c College of Chemistry and Environmental Engineering, Shenzhen University, Shenzhen, 518071, Guangdong, China; d MobiDrop (Zhejiang) Company Limited, Jiaxing, 314000, Zhejiang, China; e Agroecology Research unit, Bourgogne Franche-Comté Research Centre, National Research Institute for Agriculture, Food and the Environment, 35000, France

**Keywords:** Activated sludge, Single-cell sequencing, Antibiotic resistance genes, Plasmids, Phages

## Abstract

Wastewater treatment plants (WWTPs) represent one of biotechnology's largest and most critical applications, playing a pivotal role in environmental protection and public health. In WWTPs, activated sludge (AS) plays a major role in removing contaminants and pathogens from wastewater. While metagenomics has advanced our understanding of microbial communities, it still faces challenges in revealing the genomic heterogeneity of cells, uncovering the microbial dark matter, and establishing precise links between genetic elements and their host cells as a bulk method. These issues could be largely resolved by single-cell sequencing, which can offer unprecedented resolution to show the unique genetic information. Here we show the high-throughput single-cell sequencing to the AS microbiome. The single-amplified genomes (SAGs) of 15,110 individual cells were clustered into 2,454 SAG bins. We find that 27.5% of the genomes in the AS microbial community represent potential novel species, highlighting the presence of microbial dark matter. Furthermore, we identified 1,137 antibiotic resistance genes (ARGs), 10,450 plasmid fragments, and 1,343 phage contigs, with shared plasmid and phage groups broadly distributed among hosts, indicating a high frequency of horizontal gene transfer (HGT) within the AS microbiome. Complementary analysis using 1,529 metagenome-assembled genomes from the AS samples allowed for the taxonomic classification of 98 SAG bins, which were previously unclassified. Our study establishes the feasibility of single-cell sequencing in characterizing the AS microbiome, providing novel insights into its ecological dynamics, and deepening our understanding of HGT processes, particularly those involving ARGs. Additionally, this valuable tool could monitor the distribution, spread, and pathogenic hosts of ARGs both within AS environments and between AS and other environments, which will ultimately contribute to developing a health risk evaluation system for diverse environments within a One Health framework.

## Highlights

• This study marks the first application of high-throughput single-cell sequencing to activated sludge (AS) microbiome.
• A total of 15,110 single cells were sequenced, resulting in 2,454 single-amplified genome bins.
• The analysis detected 1,137 antibiotic resistance genes, 10,450 plasmid fragments, and 1,343 phage contigs.
• The shared relationships for plasmid (12,819) and phage (184) hosts indicate a high frequency of horizontal gene transfer.
• A set of 1,529 metagenome-assembled genomes was used to further classify 98 previously unclassified SAG bins.

## Graphical abstract

**Figure:**
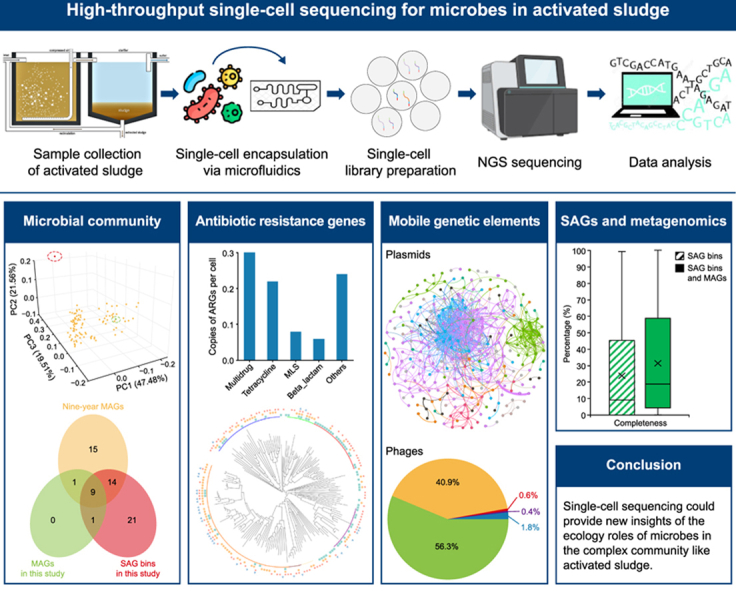

## 1 Introduction

Activated sludge (AS) is the most widely used biological treatment in wastewater treatment plants (WWTPs) for protecting the environment and human health [1]. Microbial populations in AS are abundant and diverse, and understanding what they are and what they do is key to optimizing their efficacy. WWTPs are important interfaces between humans/animals and environments [2] and are key accumulation and diffusion areas for antibiotic resistance genes (ARGs) [3] to exchange genetic material through mobile genetic elements (MGEs) [4] among complex and genetically diverse microbial communities. This emphasizes the necessity of ARG research in WWTPs based on the concept of “One Health” [5], which considers that the health of people is closely connected to the health of animals and our shared environments.

The rapid development of high-throughput sequencing and metagenomic analysis has yielded insights into the composition, function, and host relationships of microbial communities in the environment, particularly regarding the use of metagenome-assembled genomes (MAGs) as the representative genome of the same species [6]. However, as a bulk method, metagenomics still faces challenges in revealing the genomic heterogeneity of cells and hiding the discovery of microbial dark matter (MDM) since MAGs suffer from some drawbacks, including chimeric assembly artifacts, coverage bias, contamination gaps [7], and local assembly errors [8]. Additionally, correctly linking ARGs and MGEs (such as plasmids and phages) to their specific individual host cells remains a problem. For example, correctly assigning plasmids to corresponding hosts is challenging in the binning step of metagenomics because the sequence coverage of a plasmid and its host chromosome typically differ [9,10]. However, these issues could be largely resolved by single-cell sequencing, which can provide the unique genetic information of a single cell [11]. With the potential to shine a light on individual cell phenotypes, single-cell sequencing has achieved great success with mammalian cells in promoting the development of basic disciplines and underpinning processes such as the emergence of drug resistance [12,13], the evaluation of ARGs in genomes [10], and the detection of MGE–host relationships [14]. However, several factors hinder the wider adoption of this method in microbiological research. First, microbial communities are complex and diverse, requiring ultrahigh throughput to capture the diversity [15]. Second, it is challenging to amplify the whole genome with high coverage of the bacterial genome [16], especially when there is higher noise in the background, such as DNA in the extracellular matrix of AS. In addition, the cell walls of some microorganisms require rigorous lysis procedures.

Since genome amplification of a single microbial cell was achieved in 2006 for the first time [17], researchers have adopted many technologies to facilitate the development of single-cell sequencing. For example, sorting platforms, such as Raman tweezers and fluorescence-activated cell sorting, have been combined to obtain single cells at a low cost [18]. Microfluidic platforms have been integrated to achieve higher throughput for single-cell sequencing [10,19,20]. In 2022, a method called Microbeseq was proposed as a high-throughput single-cell sequencing method with strain resolution from single-cell sorting to data analysis and has been successfully applied to the human gut microbiome [21]. Microbial single-cell sequencing has wide applications in diverse environments. A study sorted and sequenced 738 single cells from the deep ocean [22] to resolve genomic information from the Deltaproteobacteria cluster SAR324. A total of 201 uncultivated archaeal and single bacterial cells from nine diverse habitats, such as hot springs and golden mines, were sequenced to explore uncharted branches of the tree of life (i.e., MDM) [23]. To investigate horizontal gene transfer (HGT) among different lineages of Prochlorococcus in the ocean, 540 single-cell genomes were sequenced [24]. To our knowledge, no study applying single-cell sequencing on AS samples has yet been conducted.

In this research, we conducted single-cell sequencing for AS samples from the Shatin (ST) WWTP, a full-scale WWTP in Hong Kong, to uncover the microbial profile of the AS community and MGE carriers (plasmids and phages) for HGT in cells of different taxonomic lineages, explore the associations of ARGs with their hosts and MGEs, investigate the complementary relationship between metagenomics and single-cell sequencing, and highlight the key technical aspects of single-cell sequencing operations for AS samples.

## 2 Materials and methods

### 2.1 Sampling, DNA extraction, and data analysis for metagenomics

ST WWTP is a secondary WWTP in Hong Kong that employs the AS biological treatment process. AS samples of 2 mL were collected from ST WWTP in May 2023 and then centrifuged at 15,000 g for 3 min (Beckman Coulter Microfuge 20R, USA) to collect pellets for deoxyribonucleic acid (DNA) extraction. Genome DNA was extracted using the FastDNA SPIN Kit for Soil (MP Biomedicals, France) according to the manufacturer's protocol. The quality and quantity of the extracted DNA were determined using a NanoDrop Spectrophotometer ND-100 (Thermo Fisher Scientific, USA) and a Qubit 2.0 fluorometer (Life Technologies, USA), respectively. The DNA sequencing of short and long reads was conducted using an Illumina sequencer from Majorbio (China) and a PromethION R10.4.1 from Oxford Nanopore (United Kingdom) in our lab at the University of Hong Kong. The sequencing data were treated with NanoPhase (version 0.2.3) [25] to generate MAGs through the hybrid assembly of short and long sequencing reads. The abundance of MAGs was calculated using CoverM (version 0.6.1) with default settings.

### 2.2 Single-cell sequencing and data analysis

Single-cell sequencing of AS samples was conducted by MobiDrop Co. Ltd. (Zhejiang) following the protocol described in a previous study [21]. The AS sample was first vortexed to homogenize the solution and release microorganisms, followed by centrifugation (1,000 rpm) and filtration (10 μm) to obtain the final cell suspension. The cell suspension was stained (Thermo Fisher LIVE/DEAD *Bac*Light Bacterial Viability Kit) to conduct cell counting, calculate cell viability, and check the homogenization condition. Then, the cell suspension was pumped into a microfluidic device to generate single cells. The whole process for single-cell sequencing included the following steps: encapsulation, lysis, amplification, tagmentation, barcoding, pooling, sequencing, and data analysis (Supplementary Material). Finally, ∼350 GB of raw sequencing data (1.1 × 9 reads) were generated and analyzed using the pipeline described in a previous publication [21] to obtain 15,110 single-amplified genomes (SAGs) and 2,454 SAG bins at the species level. An integration framework called SMAGLinker [26] was adopted to recover genomes through single-cell sequencing and metagenomics. The final collection of MAGs obtained from metagenomics contained 52 MAGs generated in this study and additional MAGs from previous works on the ST WWTP, including 920 MAGs from Wang et al. [27] and 557 MAGs from Liu et al. [7]. The taxonomies of the SAGs and MAGs were annotated using GTDB-Tk (version 2.1.1, R220) [28]. The completeness and contamination of genomes were evaluated with CheckM (version 1.2.2) [29] to distinguish genomes into high-quality (HQ) genomes, medium-quality (MQ) genomes, and low-quality (LQ) genomes [30]. Principal coordinate analysis (PCoA) was conducted using the Bray-Curtis method and visualized using R software (version 4.1.0). The phylogenetic tree was built by FastTree (version 2.1.11) [31] and visualized using the Interactive Tree of Life [32].

### 2.3 Identification of ARGs, phages, and plasmids

We identified ARGs in SAGs using the database (version 3.2.1) of ARGs online analysis pipeline (ARGs-OAP, version 3.2.2) [33] by BLAST (version 2.9.0) [34] according to the criteria of ARGs-OAP requiring 75% coverage and 80% similarity. The copy number of ARGs per cell was corrected by the coverage of ARGs and the completeness of the SAGs. Plasmids were identified using geNomad (version 1.7.4) [35] with default settings, and the length cut-off of plasmids was 1 kb [36]. The identified plasmids were aligned with a plasmid database called PLSDB (2023_11_03_v2, 59,895 records) [37] to identify plasmid fragments belonging to the same plasmid and to correct the total number of plasmids. The copy number of plasmids per cell was calculated by dividing the total number of plasmids by the total SAG number. The sequence alignment approach by BLAST (version 2.9.0) [34] was employed to identify plasmids shared among different SAG bins according to the criteria of 50% coverage and 80% identification. Phages were identified using VirSorter2 (version 2.2.4) [38] and assigned taxonomy information by geNomad (version 1.7.4) [35], with a length cut-off of 5 kb [39]. The quality of the identified phages was determined using CheckV (version 0.8.1) [40] with the checkv-db-v1.5 database. Phage groups shared by SAG bins were found using BLAST (version 2.9.0) [34] based on 50% coverage and 80% identification criteria. Fisher's exact test was conducted to filter the results (*p* < 0.05). Networks were visualized using Gephi (version 0.10.0) [41].

## 3 Results and discussion

### 3.1 The composition and diversity of the AS community revealed by single-cell sequencing

**Fig. 1:**
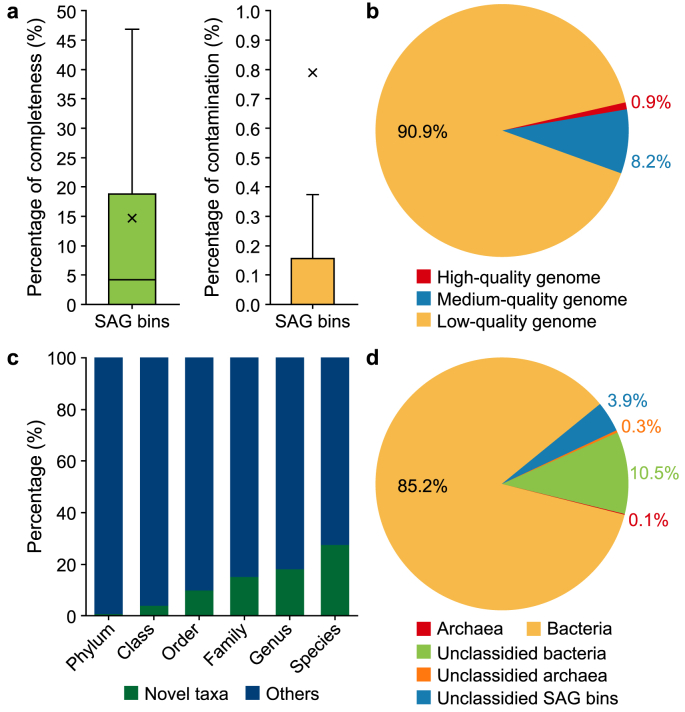
Results of single-cell sequencing. **a**, The distribution of genome information. **b**, The ratios of genome quality. **c**, Novelty percentage of SAG bins at different taxonomic levels. “Others” refers to SAG bins with known taxa and lacking the necessary information for taxonomy annotation. **d**, The taxonomic composition of microbial community for activated sludge. SAG: Single-amplified genome.

In total, ∼350 GB of raw sequencing data (1.1 ×10^9^ reads) were obtained to reveal 15,110 SAGs at the single-cell level and 2,454 SAG bins at the species level. The average contig N50, genome size, and contig number of SAG bins were 4.5 kb, 0.6 Mbp, and 266, respectively (Supplementary Material Fig. S1). Regarding the distribution of genome contamination and completeness (Fig. 1a), all genomes had low contamination (0.8% average). However, most genomes had very low completeness (14.6% average). The HQ and MQ genome percentages were 0.9% and 8.2%, respectively, and 90.9% of the SAG bins suffered low genome quality (Fig. 1b). An important objective of single-cell sequencing is to find novel genomes by providing more detailed genetic information. We investigated the novelty of SAG bins whose taxonomy annotations were available and found that 27.5% belonged to novel genomes at the species level (Fig. 1c). For example, single_bin_12_2713 showed novelty at the class level, while single_bin_12_3035 and single_bin_S_3590 had novel lineages at the order level (Supplementary Material Table S1). Results of recent studies have demonstrated the tremendous potential of single-cell sequencing to reveal MDM in WWTPs [[42], [43], [44]]. We also tried to find single-nucleotide polymorphism conditions of genomes in the AS community with MQ/HQ SAG bins, and finally, 30 substrains of 12 SAG bins were identified (Supplementary Material Table S2).

**Fig. 2:**
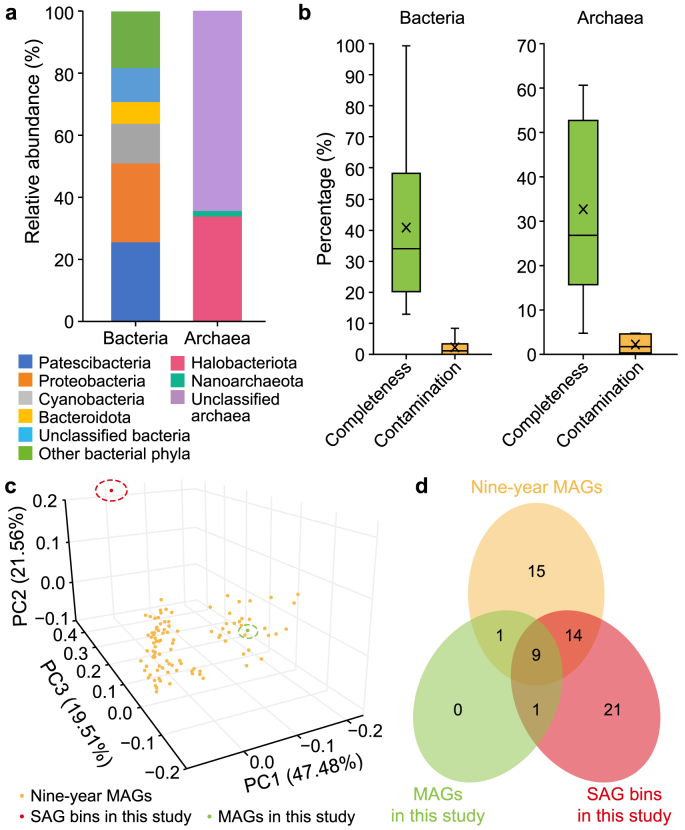
Information of annotated SAG bins and comparisons of different methods. **a**–**b**, The taxonomic composition (**a**) and genome quality (**b**) of annotated SAG bins, including 1,918 bacterial and 38 archaeal SAG bins, respectively. **c**, Principal coordinate analysis of the microbial community of activated sludge revealed by different methods, including nine-year MAGs from previous research [27] and SAG bins and MAGs generated in this study. **d**, Comparison results at the phylum level of three methods. SAG: Single-amplified genome. MAG: Metagenome-assembled genome.

We then analyzed the taxonomic composition of the SAG bins and found 1,918 bacterial and 38 archaeal SAG bins with total relative abundances of 95.7% and 0.4%, respectively. The remaining 498 SAG bins, accounting for 3.9% relative abundance in total, could not be assigned to any of the three domains due to the low completeness of the genomes (Fig. 1d). The 1,918 bacterial SAG bins were annotated with 42 phyla and were mainly distributed in the phyla Proteobacteria, Patescibacteria, and Cyanobacteria, with relative abundances of 25.6%, 25.5%, and 12.8%, respectively (Fig. 2a). The average completeness of these annotated bacterial SAG bins was ∼40%, with very low contamination of 1.0% (Fig. 2b), including some common functional groups in AS (Supplementary Material Table S3). For example, it contained three ammonia-oxidizing bacteria *g_Nitrosomonas* (average genome completeness 18.8%) and one nitrite-oxidizing bacterium *o_Nitrospiria* (genome completeness 80.7%). Some novel nitrogen-fixing microbial taxa were also detected, such as the candidate bacterial phylum Omnitrophota [45] (11 SAG bins), which has not been isolated and is poorly understood. Twenty-one SAG bins, including *g_Acinetobacter* (16), *g_Streptococcus* (3), *g_Defluviicoccus_A* (1), and *g_Moraxella* (1), were organisms associated with bulking and foaming conditions. These are all common and long-standing genera in the ST WWTP identified in our previous study [46]. Only two phyla (Halobacteriota and Nanoarchaeota) were detected for archaea, with average genome completeness and contamination of 32.7% and 2.2%, respectively (Fig. 2b).

According to the PCoA results (Fig. 2c; Supplementary Material Fig. S2a), the community composition revealed by single-cell sequencing showed an obvious discrepancy with the metagenomics in both the same sample in this study and that of previous nine-year research [27]. The dominant phyla Planctomycetota, Chloroflexota, and Actinobacteriota in ST AS [1,27,47] had very low abundance in the present single-cell sequencing results. This inconsistency between single-cell and metagenomic sequencing might be induced by insufficient genetic information, the compatibility of experiment procedures, and the characteristics of droplet-based microfluidics. In this study, many SAG bins suffered low genome completeness. They failed the taxonomy annotation due to the insufficient amplification efficiency of whole genomes and the limited sequencing ability/depth of the current short-read sequencing. The dominant microbial populations in AS may be included in these SAG bins and thus cause the difference between metagenomics and single-cell sequencing. For instance, 276 SAG bins failed the taxonomy annotation when we only used 150 GB of sequencing data for genome assembly (Supplementary Material Fig. S2b). Additionally, inconsistent experimental operations for different methods could affect the results. There is no excessive sample pretreatment for metagenomics, while the AS sample for single-cell sequencing should be homogenized and passed through the membrane to generate single cells. At the same time, the lysis reagent used in this study has been previously chosen for gut microbes [21] and might not yield good results for more complex environmental samples, such as AS. Furthermore, the droplet-based microfluidics method ensures the sequencing, assembly, and annotation of the minority, which could be ignored in the metagenomics for the AS community.

As previously noted, single-cell sequencing has the vigorous potential to mine populations with low abundance. For example, 21 bacterial phyla with a total abundance of 3.5% showed in the single-cell sequencing but failed to assemble in the MAGs collected over nine years (Fig. 2d; Supplementary Material Table S4). More importantly, these SAG bins included some recently proposed new phyla worth studying, such as the candidate phyla Margulisbacteria [48] and Riflebacteria [49]. These two phyla are not dominant in AS and contain many uncultured bacterial clades. They are thus often overlooked in bulk methods, such as metagenomics, even in long-term data over nine years, but they are successfully captured by single-cell sequencing. First identified in 2016 from MAGs of groundwater and sediment [50], Margulisbacteria was found to have 112 MAGs in GTDB (R214), and four SAG bins distributed in two classes (Marinamargulisbacteria and Termititenacia) in the single-cell sequencing of this study. As a close phylogenetic neighbor to Cyanobacteria [51], Margulisbacteria may constrain the metabolic platform for aerobic respiration and have diverse energy metabolisms with fermentation and H_2_ metabolism as central metabolic features [52]. In addition, Margulisbacteria can be selectively enriched by hydrochar during the anaerobic digestion to reach a high relative abundance of 11.6% [53]. Riflebacteria, another recently proposed phylum, was found to have 37 MAGs in GTDB (R214), and seven SAG bins of this phylum were detected in this study. It is capable of growing on carbohydrates due to fermentation or dissimilatory Fe(III) reduction [54] and contains siroheme-dependent anaerobic sulfite reductase genes for sulfite reduction [55].

### 3.2 The hosts of ARGs, plasmids, and phages revealed by single-cell sequencing

#### 3.2.1 The hosts of ARGs

**Fig. 3:**
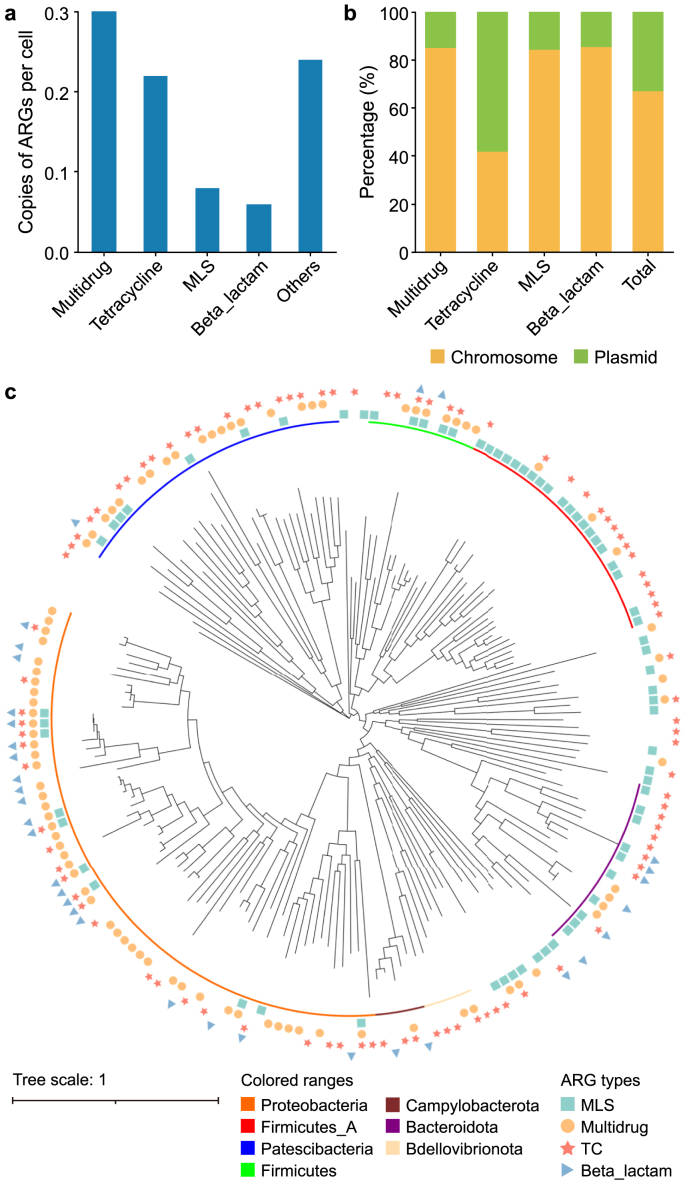
The host relationships of ARGs validated by single-cell sequencing. **a**, The results of the copy number for ARGs. **b**, The distribution of ARGs in plasmids and chromosomes in SAG bins. **c**, The phylum distribution of hosts for the top four ARGs. ARG: Antibiotic resistance gene. SAG: Single-amplified genome. MLS: Macrolide-lincosamide-streptogramin. TC: Tetracycline.

Antibiotic resistance genes are abundant in AS, but little is known about their genetic context or bacterial hosts [56]. In the present study, 1,137 ARGs were detected in 15,110 SAGs using the ARGs-OAP, corresponding to about 0.9 copies per cell. This agrees with previous studies that have revealed 0.3–0.7 copies of ARGs per cell in AS samples from the ST WWTP [57]. The four most abundant ARG classes (Fig. 3a) were multidrug (0.3 copies per cell), tetracycline (0.2 copies per cell), macrolide-lincosamide-streptogramin (MLS, 0.1 copies per cell), and beta-lactam (0.1 copies per cell). Fifteen phyla carried ARGs, and they were particularly frequently detected in the phyla Proteobacteria (41.8%), Patescibacteria (14.2%), and Firmicutes_A (11.0%), with a significant portion (34.0%) of SAGs that carried ARGs failing the taxonomy annotation (Fig. 3c; Supplementary Material Fig. S3). MLS presented more in Firmicutes_A, and the remaining three top ARGs appeared more frequently in Proteobacteria. Beta-lactam and MLS were the most conserved and variable-resistant genes, appearing in three and ten bacterial phyla, respectively. In agreement with a previously published report [58], the top four ARGs were assigned to both plasmids and chromosomes (Fig. 3b), demonstrating their mobility between plasmids and chromosomes [59]. On average, 32.8% of the ARGs occurred on plasmids. Among the top four ARGs, tetracycline had the highest occurrence in plasmids (58.2%), while beta-lactam appeared more frequently in chromosomes (85.5%). In general, the higher occurrence of ARGs in plasmids demonstrates their higher risks to environments and humans considering the high mobility of plasmids [60]. Thus, more attention should be paid to control the plasmids carrying ARGs.

The ARG profiles in the metagenomics of the same AS sample were examined. The results showed that the ARG copy number from the metagenomic results was 0.3 per cell and that the top four ARGs were beta-lactam, multidrug, sulfonamide, and MLS (Supplementary Material Fig. S4). Besides, ARGs were mainly detected in the phyla Proteobacteria, Patescibacteria, and Bacteroidota (Supplementary Material Fig. S5). Compared with metagenomics results, the amount of multidrug and tetracycline found using single-cell sequencing was higher (*p* < 0.01, Supplementary Material Fig. S6). This difference may be caused by community discrepancy since the methods for revealing the community composition have unique sample processing details. For instance, several tetracycline-resistant determinants, such as the *tet(M)* gene, are widely distributed in gram-positive species [61], and the lysis buffer used in this single-cell experiment performs better in gram-positive organisms [21]. In summary, single-cell sequencing is a powerful tool for revealing more ARG-host relationships that metagenomics might underestimate.

#### 3.2.2 The hosts of plasmids

Plasmids play a significant role in the spread of ARGs among microbes in WWTPs [62]. In the present study, 10,450 plasmid fragments were identified in 1,514 SAG bins, with an average length of 5.5 kb (Supplementary Material Fig. S7). Of the 1,514 SAG bins, 593 had taxonomy annotation and were classified into 41 phyla, with the top three phyla being Proteobacteria (193), Patescibacteria (82), and Firmicutes_A (70). This result of the host distribution is not surprising, considering that the three phyla are all dominant groups in this study. In addition, no closed circular plasmids were detected in the collection of this study. It is reasonable because achieving the complete genome of plasmids is challenging with short-read sequencing, possibly due to existing homologous sequences among plasmids and between plasmids and chromosomes in a community [63]. A total of 17.6% of plasmid fragments were identified with genes involved in conjugation, such as conjugative relaxases of MOBQ, MOBP1, and MOBV.

**Fig. 4:**
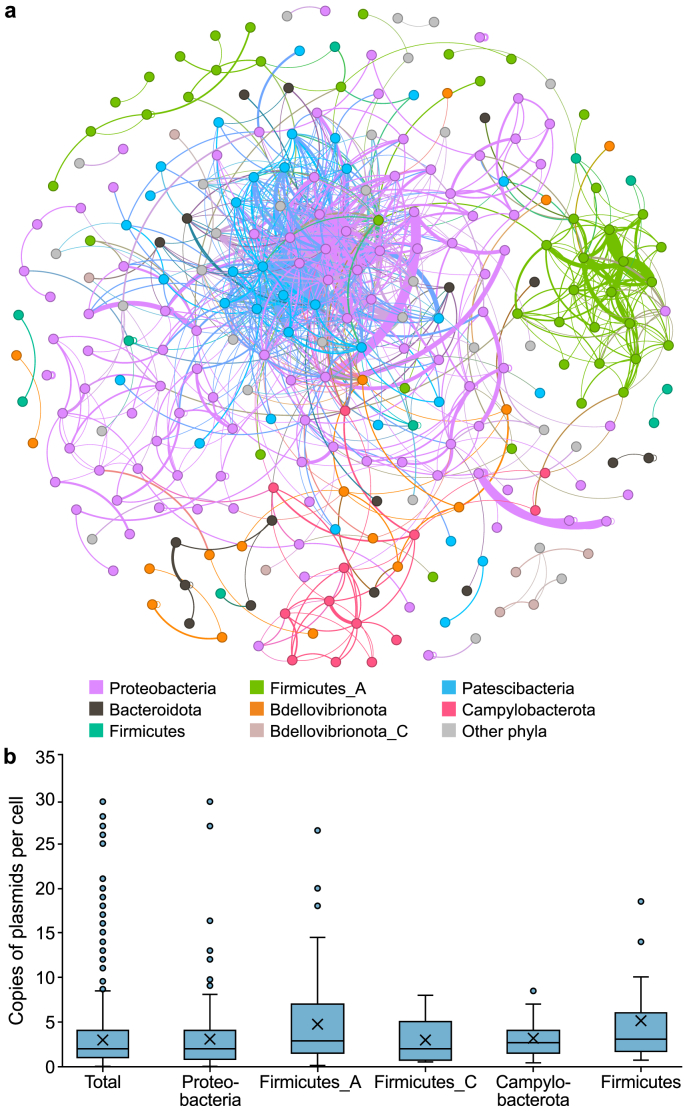
The host relationships of plasmids validated by single-cell sequencing. **a**, Network of shared plasmid groups in SAG bins. One node represents one SAG bin. **b**, The result of plasmid fragment copy number in SAG bins for different microbial phyla. SAG: Single-amplified genome.

Under such circumstances, plasmids performed active connections among SAG bins to generate up to 12,819 pairs of relationships, with 2,599 relationships created among 273 SAG bins with taxonomy annotation (Supplementary Material Table S5; Fig. 4a). These results fully demonstrate the crucial role of plasmids as typical MGEs in environments [64] and key components in HGT among microbes regarding the spread of antimicrobial resistance [65]. Our results showed that the potential HGT of plasmids mainly occurred among the hosts Proteobacteria (36.9%), Firmicutes_A (16.2%), and Patescibacteria (12.2%) with two types (Fig. 4a). For hosts like Proteobacteria, Firmicutes_A, Patescibacteria, Campylobacterota, and Bdellovibrionota, HGT mainly occurred within the same phylum, and 766 plasmids were shared by bacteria belonging to the same genus but different species. Another type of HGT occurred between different phyla. For instance, SAG bins in Patescibacteria and Cyanobacteria had higher plasmid transfer potentials than others. Their active interactions are reasonable considering the symbiotic lifestyle of Patescibacteria [66] and the special niche of predatory bacteria in the class Vampirovibrionia for Cyanobacteria [67].

Plasmids have a wide presence in microbes, and the copy number of identical plasmids in a single cell can range from one to thousands under certain circumstances [68]. Our SAG bins contained an average of 3.0 copies of plasmid fragments per cell, with a range from one to 30 copies, which was consistent with a study about gut bacteria that applied long-read PacBio sequencing to indicate that plasmids outnumbered bacterial chromosomes three to one on average [69]. Based on the current data of 491 SAG bins with taxonomy annotation (Fig. 4b), we found that Firmicutes_A had the highest average plasmid fragment number of 4.7 copies per cell, while Proteobacteria had the widest distribution of plasmid fragment numbers. This result reveals the high amount of relaxed plasmids in the AS community. In actuality, the short-read sequencing in this study could not obtain the real plasmid copy number in the cell because it generated many incomplete plasmid fragments that might belong to different parts of the same plasmid. Considering the completeness of SAG bins, the genome amplification efficiency and sequencing depth of the applied single-cell sequencing method may underestimate the existence of plasmids.

#### 3.2.3 The hosts of phages

**Fig. 5:**
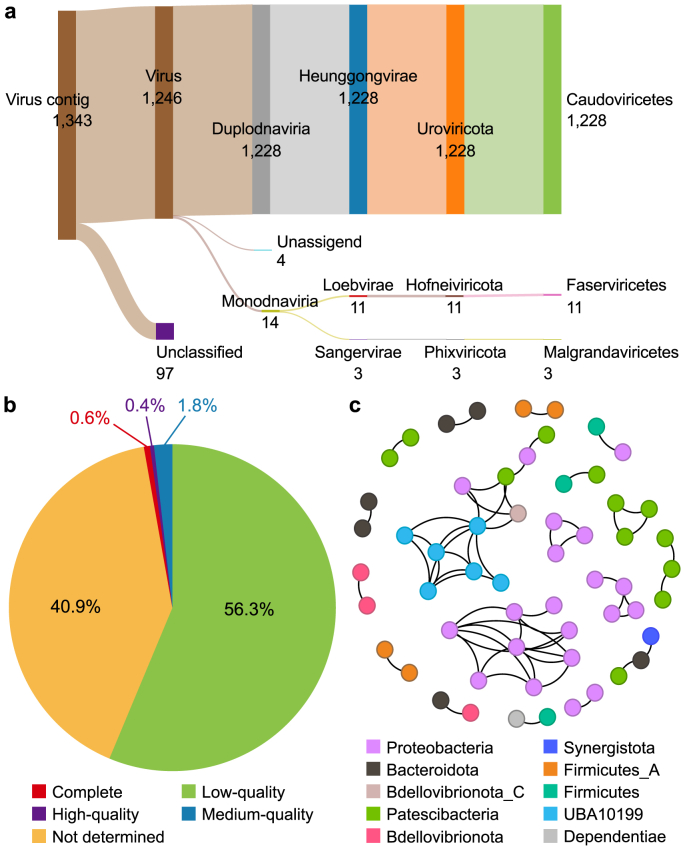
The host relationships of phages validated by single-cell sequencing. **a**, Annotation results of phages. The figure was made by SankeyMATIC. **b**, Quality results of phage contigs. The data was generated by CheckV. **c**, Network of shared phage groups in SAG bins. One node represented one SAG bin. SAG: Single-amplified genome.

A total of 1,343 phage contigs were obtained in 617 SAG bins, including 563 bacterial SAG bins, five archaeal SAG bins, and 49 unclassified SAG bins. Fig. 5a shows the phage annotation results of this study at the class level and reveals the predominant position of Caudoviricetes (1,228) in the ST WWTP. As a class of double-stranded DNA phages, Caudoviricetes is one of the most abundant and diverse phage groups in the human gut [70], while Faserviricetes and Malgrandaviricetes are filamentous single-stranded DNA phages [71]. For the taxonomy annotation of viruses, classification at higher taxonomic ranks is usually easier than at lower ranks due to the smaller interclass similarities and more abundant sequences in each class. For example, hundreds of rare genera only contain one phage in virus databases, such as the International Committee on Taxonomy of Viruses (ICTV) [72]; thus, classification at the order level or above is not as challenging as family level classification. In this study, only 5.5% of phages could be annotated at the order level, indicating the huge potential for phage mining of the AS system and the urgent demand to expand the diversity of the current database.

In this study, the top four phyla for bacterial hosts of phages were Proteobacteria (28.4%), Patescibacteria (26.1%), Firmicutes_A (9.4%), and Bacteroidota (8.1%). For the genome quality analysis, 0.6% of phage contigs had complete genomes, while 97.2% had low quality or failed the quality identification (“Not-determined”) (Fig. 5b). Recently, ARG mobilization by phages has been considered a significant mechanism responsible for the emergence of new multiresistant strains [73]. Five phages were detected to carry ARGs, including MLS (2), tetracycline (1), multidrug (1), and aminoglycoside (1). As one kind of typical MGE, phages play important roles in prokaryotic evolution [74]. Phages that could infect different hosts were recognized, and 184 groups of host–phage relationships were identified, with 59 groups showing conservatism at different taxonomic levels of the hosts (Fig. 5c; Supplementary Material Table S6). Some phages showed broad-spectrum host ranges. For example, phages of Patescibacteria could also invade the other five phyla, such as Proteobacteria and Bacteroidota. This might be due to the symbiotic lifestyle of bacteria in Patescibacteria, as they need assistance from other bacteria to grow and therefore have a higher chance of being infected by the same phage [75]. On the contrary, some phages are relatively conservative in host selection at different taxonomic levels. These include phages of single_bin_12_1138 and single_bin_12_2197 (Supplementary Material Table S6), which both belong to the same class, Alphaproteobacteria, but have different taxonomic classifications at the order level. Thus far, a wide range of studies have reported phages infecting multiple bacterial species, phyla, or even across domains [76] in diverse ecosystems, such as lakes [37] and oceans [38]. A deep understanding of the effects of phages on bacteria, especially phages with multiple hosts, is of great significance to the regulation of the microbial community in WWTPs, such as groups associated with undesirable foaming and bulking situations.

Unlike other MGEs such as plasmids, phages have extracellular phase characteristics; thus, many steps during single-cell sequencing can affect the confirmation of phage–host relationships. For instance, relationships between phages and hosts can be detected only if they have close distance to be wrapped together in the same microdroplet and generate genome amplification simultaneously. Furthermore, SAGs with enough genetic information should be recovered for the annotation of phages by increasing the sequencing quality of single-cell sequencing. Furthermore, the limitations of phage databases [77,78], especially for environmental samples like AS [79], might cause the failure of phage identification. Of course, single-cell sequencing can be combined with other technologies to uncover more phage–host associations; for example, the *in situ* method of high-throughput chromosome conformation capture technology [80,81] and the viral tagging approach to get stained viruses [82] can be used.

### 3.3 Combining single-cell sequencing and metagenomics to chart the complexity of AS microbiome

**Fig. 6:**
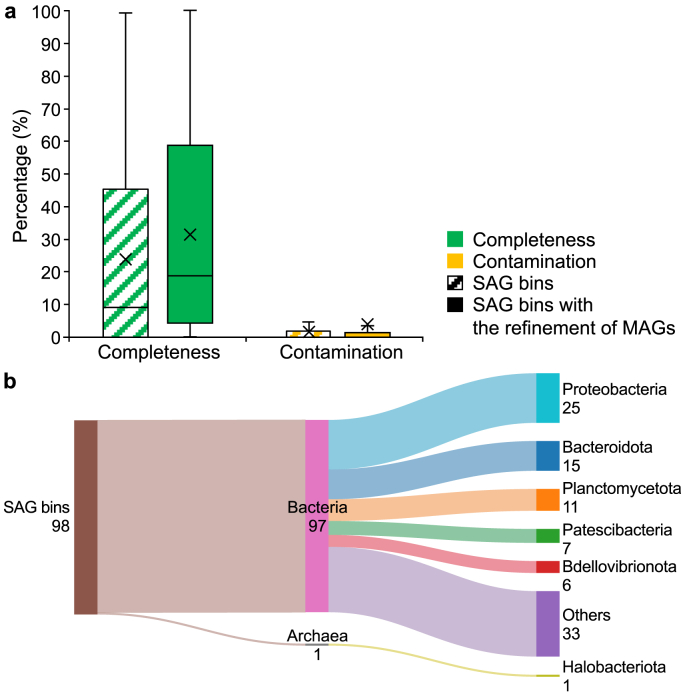
Complementary results of single-cell sequencing and metagenomics. **a**, Comparisons of genome quality of SAG bins with or without the refinement of MAGs. **b**, The taxonomy annotation for 98 SAG bins. SAG: Single-amplified genome. MAG: Metagenome-assembled genome.

A hybrid approach combining single-cell sequencing and metagenomics could yield better genome recovery and more accurate resolution of microbial populations [83,84]. We applied SMAGLinker [26] to combine SAG bins and MAGs to clarify the AS system. The genome qualty of 812 out of 2,454 SAG bins could be improved by MAGs. Fig. 6a shows that the average completeness of genomes increased from 23.8% to 31.4%, and the average contamination increased from 1.6% to 4.0%. Regarding genome quality, after genome refinement with MAGs, four more SAG bins were classified into HQ genomes, and 29 genomes could be improved to MQ genomes. More HQ genomes could have been recovered but failed due to the high genome contamination. Therefore, how to utilize algorithms to enhance genome completeness and ensure a low contamination level is a crucial technical question for subsequent pipeline development. Moreover, 98 SAG bins (Fig. 6b) that were annotated as “unclassified” before genome refinement with MAGs were successfully assigned to 20 phyla, with the top three phyla being Proteobacteria (25), Bacteroidota (15), and Planctomycetota (11), and all of them were dominant groups in AS. However, the abundance of these newly resolved SAG bins was low, and they only increased the resolved abundance percentage of AS by 1.7%.

We then explored the host relationships of ARGs, plasmids, and phages for the refined SAG bins. Compared with the results of original SAG bins, we found an extra 22 ARGs, 60 plasmid fragments, and 51 phages. Distributed in 17 refined SAG bins, the majority of 22 ARGs belonged to the multidrug type and only one ARG appeared in the plasmid. In addition, over 96.1% of the newly identified phages belonged to the class Caudoviricetes. All of the above results demonstrate the complementary roles of MAGs in providing valuable genome information to SAGs. Correspondingly, SAGs can determine the correct assembly results and provide more details about the heterogeneity among different cells for MAGs. Combining these two sequencing approaches can comprehensively reveal the microbiome community from both macro and micro perspectives. At the same time, optimized algorithms and powerful bioinformatic tools should be developed to facilitate this procedure, as the current SMAGLinker tool is insufficient to improve the genome quality.

### 3.4 The key insights of quality control for single-cell sequencing of AS microbiome

Activated sludge is a complex ecosystem with a high biomass of (4–6) × 10^23^ bacteria globally [85]. Unlike bulk-DNA sequencing such as metagenomics, the high biodiversity and heterogeneity of AS pose challenges in every step of single-cell sequencing. Thus, quality control should be done to avoid experimental bias and ensure the reliability of the final results. For instance, microbes that have difficulty in forming flocs and exist in the supernatant will be significantly lost during sedimentation. Additionly, the dense floc structure of AS may make it difficult for some bacteria to form the single-cell state and thus make it hard to detect or cause the cross-contamination. Therefore, well-homogenized suspension is particularly important in the single-cell sequencing of AS. There are many homogenization methods for AS samples, such as mechanical stirring [86], ultrasonication [87], and pressure [88]. Fluorescent staining and microscopy were combined to confirm the homogeneity of samples. At the same time, overly aggressive homogenization methods should be avoided because the vital relationships derived from physically tightly coupled interactions, including symbiosis, predation, and parasitism, may be destroyed during the process and thus cannot be identified.

In this study, three groups of microbes were more likely to be observed in single-cell sequencing. The first group of microorganisms such as Proteobacteria, occupied a high abundance in AS and thus had a higher chance of being detected. The second group included gram-positive microorganisms, for instance Firmicutes, because the lysis system used in this experiment performs better for gram-positive than gram-negative microbes [21]. In addition, microbes with small genome sizes are more easily amplified and assembled. For example, bacteria belonging to Patescibacteria and UBA10199 accounted for a greater portion of our results. Compared to human-related bacterial studies, the significance of single-cell sequencing at the strain level on environmental ecosystems like AS needs to be further explored. To illustrate, researchers can explore the significance of metabolic variabilities and evolutionary relationships in different strains of microbial functional groups that can remove nitrogen and phosphorus from wastewater.

Apart from sample pretreatment, the technological improvement of single-cell sequencing is also crucial. Compared with metagenomics, single-cell sequencing in this research showed advantages in genomic heterogeneity mining, minority population verification, and host–MGEs relationship identification. However, the fact that many SAGs were of poor quality and lacked enough genetic information limited the exploration of more significant findings. Genome quality is of great significance because HQ or complete genomes with less contamination are essential for understanding the phylogeny and function of microbes, especially the uncultured ones in complex microbial ecosystems. Thus, more effort should be placed on improving the resolution of single-cell sequencing on AS using multiple strategies, such as increasing the sequencing depth, finding amplification enzymes with higher efficiency, and applying long-read sequencing to recover genomes. In 2023, researchers developed a supreme enzyme, HotJa Phi29 DNA, by further protein and process engineering [89] to show significantly improvement in single-cell genome amplification of 99.8% coverage. The rapid development and high accuracy (Q20) of long-read sequencers, such as Oxford Nanopore [25] and Pacific Biosciences [90], in recent years, have helped metagenomics enter a new era of genome quality improvement and will open doors to single-cell sequencing, such as getting the complete genome of the chromosomes and MGEs of plasmids and phages. With the continuous development and refinement of single-cell technologies, it is important to combine multiple omics technologies in future environmental studies (e.g., single-cell transcriptome such as BacDrop [20], and spatial omics) to comprehensively reveal the dynamics of microbial populations during wastewater treatment and responses to the disturbance. These technologies can help engineers understand the changing trends of microbial communities with timely control, which is of great significance for engineered microbial communities like AS.

## 4 Conclusions

The present study validated the application of high-throughput single-cell sequencing in the field of AS using 15,110 single cells to reveal fresh perspectives. The results showed that single-cell sequencing revealed a community composition shift with metagenomic results and identified SAG bins mainly belonging to the phyla Proteobacteria, Patescibacteria, and Cyanobacteria, with 27.5% having novel genomes at the species level. Additionally, 1,137 ARGs, 10,450 plasmid fragments, and 1,343 phage contigs were confirmed to explore HGT conditions among different hosts. Although a hybrid approach integrating metagenomics and single-cell sequencing could provide more genome information, many novel insights into genomes might be hidden due to the limited genome size and quality of SAG bins in this study. For wider applications in the future, single-cell sequencing should combine other technologies, such as the long-read sequencing and high-performance enzymes, to further promote the genome quality of SAGs for better charting of ecological roles for different microbiome.

## CRediT authorship contribution statement

**Yulin Zhang:** Writing - Original Draft, Methodology, Conceptualization, Investigation. **Bingjie Xue:** Writing - Review & Editing, Investigation. **Yanping Mao:** Writing - Review & Editing, Resources. **Xi Chen:** Writing - Review & Editing, Data curation. **Weifu Yan:** Writing - Review & Editing, Investigation. **Yanren Wang:** Writing - Review & Editing. **Yulin Wang:** Writing - Review & Editing, Resources. **Lei Liu:** Writing - Review & Editing, Resources. **Jiale Yu:** Methodology. **Xiaojin Zhang:** Methodology. **Shan Chao:** Methodology. **Edward Topp:** Writing - Review & Editing. **Wenshan Zheng:** Writing - Review & Editing, Methodology. **Tong Zhang:** Writing - Review & Editing, Methodology, Conceptualization.

## Declaration of competing interest

The authors declare that they have no known competing financial interests or personal relationships that could have appeared to influence the work reported in this paper.
