# Supplementary material for: High-throughput single-cell sequencing of activated sludge microbiome
Source: Environ Sci Ecotechnol. 2024 Sep 12;23:100493. doi: 10.1016/j.ese.2024.100493 (PMC11490935; doi:10.1016/j.ese.2024.100493)
Supplement: Multimedia component 2 [file mmc2.docx]

**High-Throughput Single-Cell Sequencing of Activated Sludge Microbiome**

Yulin Zhang ^a^, Bingjie, Xue ^a, b, c^, Yanping Mao ^c^, Xi Chen ^a^, Weifu Yan ^a^, Yanren Wang ^a^, Yulin Wang ^a^, Lei Liu  ^a^, Jiale Yu ^d^, Xiaojin Zhang ^d^, Shan Chao ^d^, Edward Topp ^e^, Wenshan Zheng ^d^, Tong Zhang ^a, b^*

^a^ Environmental Microbiome Engineering and Biotechnology Lab, Department of Civil Engineering, The University of Hong Kong, Pokfulam Road, Hong Kong, 999077, China

^b^ School of Public Health, The University of Hong Kong, Pokfulam Road, Hong Kong, 999077, China

^c^ College of Chemistry and Environmental Engineering, Shenzhen University, Shenzhen, 518071, Guangdong, China

^d^ MobiDrop (Zhejiang) Company Limited, Jiaxing, 314000, Zhejiang, China

^e^ Agroecology Research unit, Bourgogne Franche-Comté Research Centre, National Research Institute for Agriculture, Food and the Environment, 35000, France

*Corresponding author. Phone: +852-28578551. Fax: +852-25595337. E-mail: [zhangt@hku.hk](mailto:zhangt@hku.hk).

Physical mailing address: HW 6-31, The University of Hong Kong, Pokfulam, Hong Kong

**1 Experimental process demonstration of single-cell sequencing**

The following steps were summarized according to the published reference [1] and protocol of Majorbio company (China).

1) Microbial Suspension Preparation: Following the preparation of samples into microbial suspensions, microbial viability was assessed using Live/Dead staining.

2) Microbial Lysis within Droplets: Employ lysis solution to break down microorganisms, releasing microbial DNA.

3) Whole-Genome Amplification: Utilize Multiple Displacement Amplification (MDA) to amplify the DNA.

4) DNA Fragmentation: Fragment the DNA within the droplets.

5) DNA Capture and PCR Amplification: Label the DNA within the droplets to capture it and perform amplification.

6) Droplet Disruption: Break the droplets.

7) Library Construction: Purify enriched DNA using magnetic beads, add sequencing primers and indexes to both ends of the purified DNA through PCR amplification, and perform DNA enrichment.

8) Illumina Sequencing: The final library was coupled with oligos on the Flowcell, generating millions of clusters through bridge amplification. Fluorescently labeled dNTPs were utilized for DNA synthesis, with only one DNA strand being synthesized per cycle.4 Images were captured to read the sequence of each synthesized DNA fragment, followed by the analysis of fluorescent signals to deduce the sequence of template DNA fragments.

**2 Bioinformatics analysis process of single-cell sequencing**

**2.1 Raw data process**

After filter according to the barcode whitelist, the raw fastq files were splited into three files: the barcode fastq files, the R1-files of insert sequence and the R2-files of the insert sequence. The R1-files and R2-files were processed by fastp software to get clean fastq files which used in co-assemble process.

**2.2 Co-assemble of microbe genomes at species level**

Assemble the similar SAGs. And find the SAGs which similar to the contigs that assembled previously. Keep running the process until relatively complete genome sequences were reached. This co-assemble method was used to get the genome of a microbe at species level. Then checkM was used to estimate the completeness and contamination of each microbe genomes and quality rank was assigned. For the genomes with high or medium quality rank, the GTDB-Tk and Refseq were used to annotate at species level.

**2.3 Assemble of strain-resolved genomes**

For the medium-/high-quality SAGs, re-map the reads of SAGs to the microbe genome at species level which the SAGs belong to achieve SNP information. Use the SNP information to cluster the SAGs to identify possible strains. Use a certain cluster of SAGs to assemble the genome of the strain.

**Figures**

**Figure S1** The assembly results of SAG bins for single-cell sequencing.


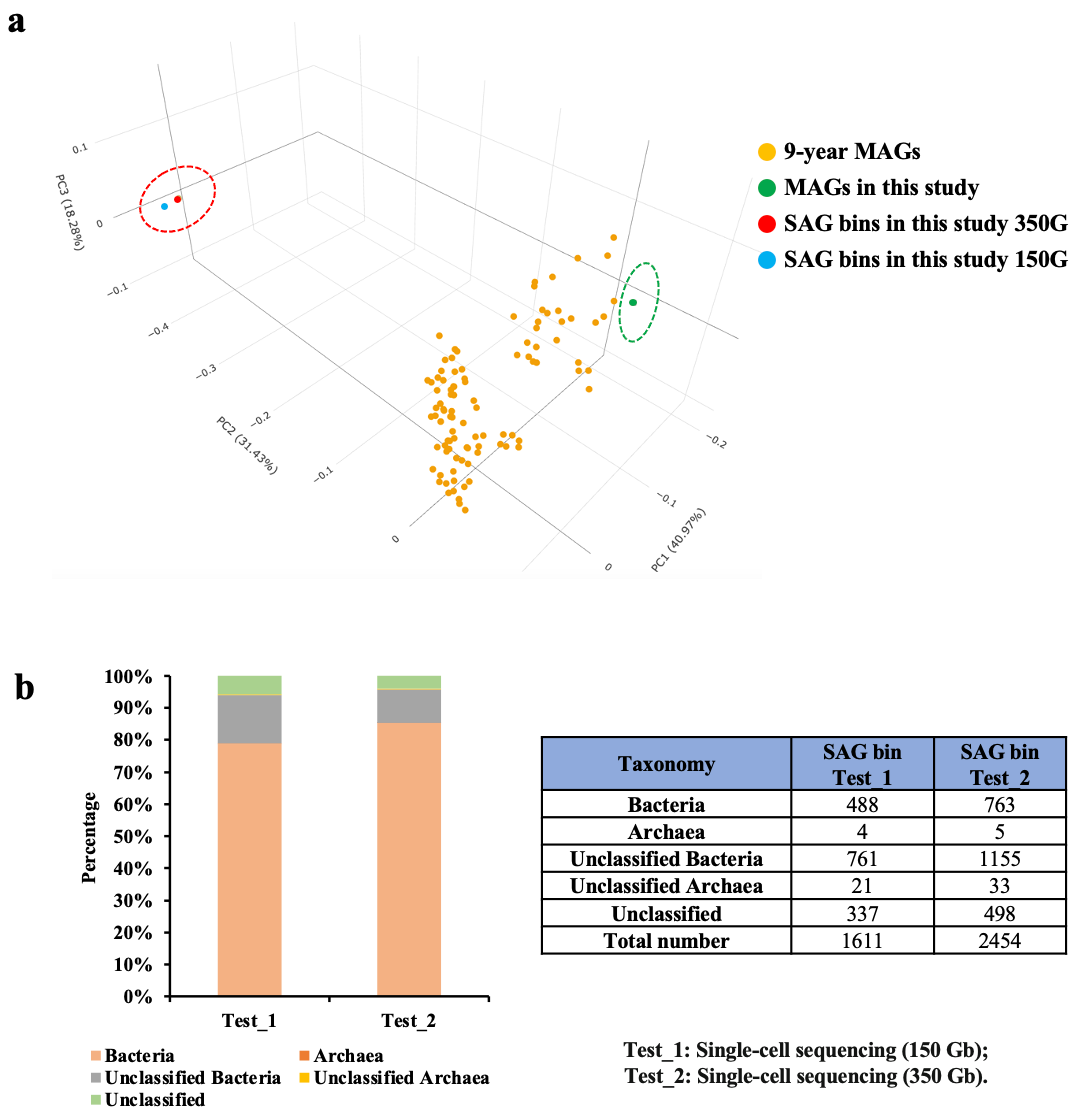


**Figure S2** The community composition of AS revealed by the single-cell sequencing and metagenomics. a) The PCoA results of the single-cell sequencing and metagenomics. b) The abundance of taxonomic composition for single-cell sequencing.

**Figure S3** The distribution of ARG type for AS community revealed by single-cell sequencing at the SAG level.


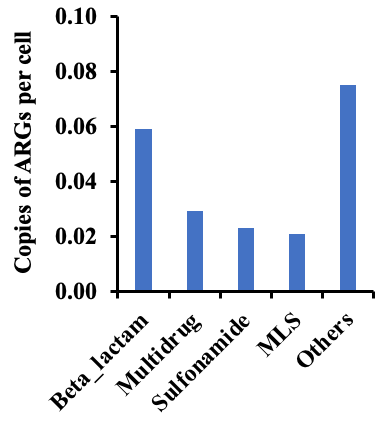


**Figure S4** ARG distribution of the AS community revealed by metagenomics *via* ARGs-OAP.


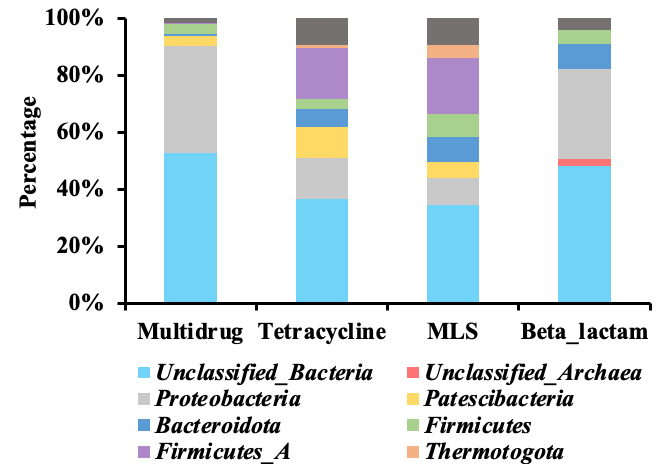


**Figure S5** The distribution of ARG hosts at the phylum level for the AS community revealed by metagenomics *via* ARGs-OAP.

**Figure S6** One-Factor ANOVA of single-cell sequencing and metagenomics for multidrug and tetracycline. **p*<0.05, ***p*<0.01, ****p*<0.001.

**Figure S7** The length distribution for plasmids validated by single-cell sequencing.

**References**

1. Zheng, W., et al., *High-throughput, single-microbe genomics with strain resolution, applied to a human gut microbiome.* Science, 2022. **376**(6597): p. eabm1483.
